# Knowledge of mother-to-child transmission of HIV among women living with HIV in Malawi

**DOI:** 10.1177/09564624241246297

**Published:** 2024-04-26

**Authors:** Roger Antabe, Yujiro Sano, Joseph Kangmennaang

**Affiliations:** 1 Department of Health and Society, 33530 University of Toronto Scarborough, Toronto, ON, Canada; 2 Department of Sociology and Anthropology, Nipissing University, North Bay, ON, Canada; 3 School of Kinesiology and Health Studies, 4257 Queen’s University, Kingston, ON, Canada

**Keywords:** Mother-to-child transmission, HIV, knowledge, Malawi, sub-Saharan Africa

## Abstract

**Objectives:**

To reduce the incidence of mother-to-child transmission (MTCT) of HIV in Malawi, interventions have been created for women to be informed about the MTCT of HIV and for women living with HIV to be entered into the HIV care cascade to ensure safer deliveries. Our study aimed to examine the effectiveness of these strategies by exploring the determinants of adequate knowledge of MTCT of HIV among women living with HIV in Malawi.

**Methods:**

We used the 2015-16 Malawi Demographic and Health Survey data and applied logistics regression analysis to explore the determinants of adequate knowledge of MTCT of HIV among women living with HIV.

**Results:**

Our findings estimated that 75% of women living with HIV possessed adequate knowledge of MTCT of HIV. We also found that compared to those with no formal education, women with primary education (OR = 1.88, 95% CI = 1.04, 3.41) and secondary education or higher (OR = 2.61, 95% CI = 1.21, 5.62) were more likely to have adequate knowledge of MTCT of HIV. Furthermore, women who were resident in rural areas (OR = 2.97, 95% CI = 1.58, 5.57), were more likely to have adequate knowledge of MTCT of HIV relative to those in urban areas. Finally, women who had adequate HIV knowledge (OR = 1.85, 95% CI = 1.19, 2.89) and those who rejected the endorsement of HIV stigma and discrimination (OR = 2.30, 95% CI = 1.39, 3.81) were more likely to have adequate knowledge about the MTCT of HIV.

**Conclusion:**

Based on our findings, there is an urgent need to offer women living with HIV in Malawi the opportunity to increase their knowledge of MTCT of HIV if the country is to make progress towards the elimination of MTCT of HIV as part of the overall strategy to contain new HIV infections in the country.

## Introduction

New infections of the human immunodeficiency virus (HIV) in Malawi continue to undermine the country’s progress towards reducing HIV prevalence below endemic levels. Malawi’s adult HIV prevalence in 2022 was 7.1%, with the Malawi National AIDS Commission (NAC) estimating that there were a total of 15,700 new HIV infections across all ages.^1^ About 17% of these new infections in 2022 were among children aged 0-14 years in the country, bringing into focus the role of mother-to-child transmission (MTCT) of HIV.^1^ MTCT of HIV, also called vertical transmission of HIV, occurs when an expectant mother or a postnatal mother who is living with HIV transmits the virus to their children during pregnancy, labour/delivery or through breastfeeding.^2^ The risk of MTCT of HIV in Malawi is particularly concerning as the HIV prevalence among pregnant women has been estimated at 10% to 30%, with some HIV-positive women being unaware of their HIV serostatus or initiating treatment late into their pregnancy.^3,4^

In line with the recommendations by the World Health Organization (WHO) and the Joint United Nations Programme on HIV/AIDS (UNAIDS) on MTCT of HIV, there have been initiatives by the Government of Malawi to eliminate all vertical transmissions of HIV.^5^ In the third edition of the *Guidelines for the Clinical Management of HIV Malawi*, published by the Malawi Ministry of Health, the document endorsed a four-pronged approach to addressing MTCT of HIV, which included emphasis on the primary prevention of HIV infection among parents; prevention of unintended pregnancies among women who are living with HIV; preventing transmission of HIV from HIV positive parents to their children; and providing care, treatment and support for women living with HIV, including their children and families.^6^ Central to this document and the four-pronged approach is making women living with HIV aware of the risk of vertical transmission of HIV and initiating steps, including enrolling them into antiretroviral therapy (ART) and providing them with clinically assisted births.^6^ These approaches are integrated into maternal, newborn and child health programs as recommended by the WHO.^7^

The importance of enhancing awareness and knowledge of MTCT of HIV among women living with HIV is critical to transitioning them into the HIV care cascade that guarantees them safer delivery. For instance, the enrolment of pregnant women and breastfeeding women living with HIV into the Option B+ strategy (putting them on lifelong antiretroviral treatment regardless of their Clusters of Differentiation 4 (CD4) count) as part of PMTCT in Malawi resulted in a substantial increase in the number of women initiating ART, a decline in early infant transmission of HIV at 3.7%, an increased knowledge of HIV status and early initiation of ART during pregnancy.^4,8,9^ As part of prevention of MTCT of HIV strategies, creating awareness among women living with HIV is further hinged on the WHO’s observation that the absence of an MTCT of HIV policy response increases the risk of vertical transmission by 15%-45%.^2,10^

Additional attempts to increase knowledge of MTCT of HIV among women in Malawi has been undertaken by the government. For instance, the country’s success as the first globally to transition the prevention of MTCT to Option B+ did not only involve increasing HIV testing for expectant and lactating mothers and enrolling those testing positive to lifelong antiretroviral treatment, but also targeting such women with clinical information on MTCT of HIV and HIV transmission.^4,11^ Additionally, in the National HIV Prevention Strategy 2015-2020, extra efforts were outlined under the prevention of MTCT of HIV by including social behaviour change specifically targeted at high-risk women including those aged 15–24 years, women in relationships, and female sex workers. Furthermore, women living with HIV were to be given additional care and counselling while ensuring their unhindered access to contraceptives in reducing unintended pregnancies.^12^

Given the crucial role of women’s knowledge of MTCT of HIV to the elimination of vertical transmission, studies elsewhere in sub-Saharan Africa (SSA) have explored the prevalence and determinants of MTCT of HIV knowledge among women. For instance, in a cross-sectional study among 33 countries in SSA, Teshale and colleagues^13^ found that only 56% of women were knowledgeable about MTCT of HIV and that being older, having higher educational attainment, coming from a richer household, having a birth parity of one or more and being exposed to mass media were associated with a higher odds of having knowledge about MTCT of HIV. Among women attending antenatal care (ANC) in Kombolcha Town in Ethiopia, 61% of them were reported to have had adequate knowledge about MTCT Option B+ and adequate knowledge of MTCT Option B+ was also associated with age, educational attainment, ANC visits and birth parity.^14^ At the national level in Ethiopia, it was also established that 86% of women in their reproductive ages were knowledgeable about MTCT of HIV with the richest, those exposed to mass media, those with comprehensive knowledge of HIV and knowing where to get an HIV test being more likely to have knowledge about MTCT of HIV.^15^ Finally, among women in the Ndola district in Zambia, only 58% of women were found to have possessed adequate knowledge about vertical HIV transmission.^16^

While findings from these earlier studies are useful for health and HIV policy relating to MTCT of HIV, they are exclusively focused on all women of reproductive age. This limits our understanding of the effectiveness of initiatives that are mostly targeted at increasing awareness of MTCT of HIV among women living with HIV. Our study, therefore, sought to explore the prevalence and determinants of knowledge of MTCT of HIV among Malawian women living with HIV. This is particularly important as an earlier study elsewhere in Cameroon has linked such knowledge to the timely initiation and uptake of antenatal care services that help reduce the chances of vertical transmission.^17^ Similarly, Tippett Barr et al.^9^ found in Malawi that women living with HIV may be initiating ART at later stages during their postpartum periods, although this tends to increase the risk of MTCT of HIV. Emphasising the need for timely initiation of treatment for women living with HIV during pregnancy, van Lettow et al.^18^ further observed that women who missed any of the recommended steps for the prevention of MTCT of HIV increased their likelihood of vertical transmission. These revelations underscore the critical role of women living with HIV’s knowledge of MTCT of HIV to their timely initiation of treatment during pregnancy.

## Methods

To understand the prevalence and determinants of knowledge of MTCT of HIV among women living with HIV, we used the 2015-16 Malawi Demographic and Health Survey (MDHS) that was implemented by the National Statistical Office, with technical assistance from ICF. The MDHS provides current estimates of demographic and health indicators including women’s knowledge of MTCT of HIV. The MDHS employed a multi-staged sampling framework where systematic sampling with probably to size was applied for identify enumeration areas from which households were chosen. There were 25,146 women identified for individual interviews. Out of these participants, interviews were completed with 24,562 women, with a response rate of 98%. The MDHS included HIV prevalence testing for women aged 15-49. Based on this information, we narrowed down our analytical sample to 858 women who tested positive for HIV.

### Measures

The dependent variable captures whether women have adequate knowledge about MTCT of HIV. We used three binary questions to construct this variable, asking respondents whether HIV can be transmitted from a mother to her baby (1) during pregnancy, (2) during delivery, and (3) by breastfeeding. Specifically, respondents were coded “yes” if they answered all three questions correctly and “no” otherwise (o = no; 1 = yes). Informed by a review of the literature, we introduced three sets of independent variables, namely socioeconomic, demographic, and HIV Prevention Knowledge variables. There are two socioeconomic variables such as education (0 = no education; 1 = primary education; 2 = secondary/higher education) and household wealth (0 = poorest; 1 = poorer; 2 = middle; 3 = richer; 4 = richest). We further considered four demographic variables, namely place of residence (0 = urban; 1 = rural), region of residence (0 = Northern; 1 = Central; 2 = Southern), marital status (0 = never married; 1 = currently married; 2 = formerly married), and age of respondents (measured in completed years). Two HIV Prevention Knowledge variables include adequate HIV knowledge (0 = no; 1 = yes) and HIV-related stigma and discrimination (0 = yes; 1 = no). For adequate HIV knowledge, respondents were asked whether they believe that the risk of getting HIV can be reduced through (1) not having sex at all, (2) always using condoms during sex, and (3) having one sex partner only. We summarized these responses where respondents were coded “yes” if they answered all three questions correctly and “no” otherwise (o = no; 1 = yes). For HIV-related stigma and discrimination, we used two questions, asking respondents whether (1) they would buy vegetables from vendors with HIV and (2) children with HIV should be allowed to attend school with children with HIV. When respondents answered “no” to at least one of these questions, we coded them as “yes” and “no” otherwise (0 = yes; 1 = no).

### Statistical analysis

We employed descriptive and regression analysis. First, we use descriptive statistics to understand the characteristics of the analytical sample. Second, we use logistic regression analysis to explore factors associated with having adequate knowledge about MTCT of HIV. We calculated both unadjusted and adjusted estimates. For adjusted estimates, we simultaneously accounted for socioeconomic, demographic, and HIV prevention knowledge factors. Results were shown with odds ratios (ORs). ORs larger than 1 indicate that women are more likely to have adequate knowledge about MTCT of HIV, while those smaller than 1 point to lower odds of having adequate knowledge. All analyses used STATA 17 (State Corp, College Station, TX, USA). The ‘svy’ function is applied in statistical analysis to adjust for the cluster sampling design as well as sampling weights.

## Results

Table 1 shows sample characteristics. In terms of education, about three in five respondents (59%) had primary education while 14% did not have any formal education. The majority lived in rural areas (70%) as well as Southern Region (68%). We also observed the mean age of respondents was 33 years old, where as 71% had adequate HIV knowledge and 15% endorsed HIV-related stigma and discrimination. Overall, we found that 75% of women living with HIV had adequate knowledge about MTCT of HIV.

**Table 1.** Sample characteristics (*n* = 858).

	Percentage
Adequate knowledge about MTCT of HIV/AIDS	
No	25
Yes	75
Education	
No education	14
Primary education	59
Secondary/higher education	27
Household wealth	
Poorest	16
Poorer	14
Middle	18
Richer	20
Richest	32
Place of residence	
Urban	30
Rural	70
Region of residence	
Northern	6
Central	26
Southern	68
Marital status	
Never married	10
Currently married	62
Formerly married	28
Age^a^	33
Adequate HIV knowledge	
No	29
Yes	71
HIV-related stigma and discrimination	
Yes	15
No	85
Total	100

^a^Mean reported for age.

Table 2 shows unadjusted logistic regression results. We found that women with primary (OR = 2.00, *p* < .01) and secondary/higher education (OR = 2.29, *p* < .01) were more likely to have adequate knowledge about MTCT of HIV in contrast to those with no education. Similarly, rural women were more likely to have adequate knowledge about MTCT of HIV in contrast to their urban counterparts (OR = 1.68, *p* < .05). Formerly-married women were also more likely to have adequate knowledge about MTCT of HIV compared to never-married women (OR = 2.35, *p* < .05). Moreover, compared to those without adequate knowledge, women with adequate HIV knowledge were more likely to have adequate knowledge about MTCT of HIV (OR = 2.48, *p* < .01). Women without HIV-related stigma and discrimination were more likely to have adequate knowledge about MTCT of HIV in comparison with those with such stigma and discrimination (OR = 2.66, *p* < .01).

**Table 2.** Adjusted and adjusted logit models predicting adequate knowledge about MTCT of HIV/AIDS among HIV + women in Malawi.

	Unadjusted			Adjusted		
	OR	95% CI		OR	95% CI	
Education						
No education	1.00			1.00		
Primary education	2.00***	1.20	3.34	1.88**	1.04	3.41
Secondary/higher education	2.29***	1.23	4.26	2.61**	1.21	5.62
Household wealth						
Poorest	1.00			1.00		
Poorer	1.17	0.66	2.06	1.32	0.72	2.43
Middle	1.07	0.61	1.89	1.36	0.73	2.54
Richer	0.95	0.50	1.82	1.47	0.73	2.94
Richest	1.20	0.61	2.34	2.19	0.84	5.66
Place of residence						
Urban	1.00			1.00		
Rural	1.68**	1.11	2.53	2.97***	1.58	5.57
Region of residence						
Northern	1.00			1.00		
Central	1.63	0.82	3.24	1.89*	0.94	3.81
Southern	1.52	0.79	2.93	1.76*	0.90	3.43
Marital status						
Never married	1.00			1.00		
Currently married	1.53	0.76	3.10	1.60	0.68	3.79
Formerly married	2.35**	1.03	5.39	2.66	0.96	7.39
Age	1.00	0.98	1.02	0.98	0.95	1.01
Adequate HIV knowledge						
No	1.00			1.00		
Yes	2.48***	1.61	3.84	1.85**	1.19	2.89
HIV-related stigma and discrimination						
Yes	1.00			1.00		
No	2.66***	1.58	4.47	2.30***	1.39	3.81

*p < .1, **p < .05, ***p < .001.

Table 2 also shows adjusted logistic regression results, indicating that adjusted results are largely consistent with unadjusted ones, except that marital status became no longer significant. For example, we found that women with primary (OR = 1.88, *p* < .05) and secondary/higher education (OR = 2.61, *p* < .05) were more likely to have adequate knowledge about MTCT of HIV in contrast to those with no education. Similarly, rural women were more likely to have adequate knowledge about MTCT of HIV in contrast to their urban counterparts (OR = 2.97, *p* < .01). Moreover, compared to those without adequate knowledge, women with adequate HIV knowledge were more likely to have adequate knowledge about MTCT of HIV (OR = 1.85, *p* < .01). Women without HIV-related stigma and discrimination were more likely to have adequate knowledge about MTCT of HIV in comparison with those with such stigma and discrimination (OR = 2.30, *p* < .001).

## Discussion

MTCT of HIV in Malawi has remained a persistent public health issue contributing to the burden of HIV among children. In 2022, for instance, MTCT of HIV accounted for 17% of overall new HIV cases in the country.^1^ While the policy response has been, among other things, to increase awareness of MTCT of HIV among women in Malawi as a first step to getting HIV positive expectant mothers into the HIV care cascade, we still do not know the prevalence and determinants of knowledge of MTCT of HIV among women living with HIV. This is particularly concerning as women living with HIV in Malawi continue to be the main targets of prevention strategies. To contribute to the HIV policy and literature, our study specifically explored the prevalence and determinants of knowledge of MTCT of HIV among women living with HIV in Malawi. Our findings estimated that 75% of women living with HIV in Malawi have adequate knowledge of MTCT of HIV. This level of knowledge of MTCT of HIV among HIV positive women in Malawi may be higher than what has previously been reported among women of reproductive age in the settings of SSA and elsewhere. For instance, in a study across 33 countries, only 56% of women of reproductive age were reported to have had adequate knowledge about MTCT of HIV.^19^ Similarly, among pregnant women attending ANC in the Ndola district in Zambia, only 57% were found to possess adequate knowledge about MTCT of HIV.^16^ This finding supports the observation by Tippett-Barr et al.^9^ and Kim et al.^4^ that Malawi has adopted innovative approaches to addressing MTCT of HIV. That is, being the first country globally to transition the prevention of MTCT of HIV to Option B+, there was improved attention to testing all expectant and lactating women, and those testing positive for HIV were prioritized to receive lifelong treatment of antiretroviral treatment and clinical information on the vertical transmission of HIV.^4,20^ While this initiative has reported positive benefits for addressing MCTC of HIV in the country, there is still the need for additional efforts to target more women living with HIV with information about their heightened risk of vertical transmission of HIV and the mechanisms through which MTCT of HIV can be prevented.

We also observed that educational attainment was associated with knowledge of MTCT of HIV where women living with HIV who had completed primary or secondary levels of education were more likely to possess adequate knowledge of MTCT of HIV relative to their counterparts with no formal education. This finding is consistent with the existing literature that suggests higher educational attainment provides a useful medium for people to become more assertive about their health and health care needs, thus, putting in extra efforts to access health information that helps protect their health.^21–24^ In this regard, it is also possible that women living with HIV in Malawi with formal education are in a better position to search for, read and digest health information related to HIV and MTCT of HIV relative to their counterparts who may need some assistance to do so. Among women attending ANC in Kombolcha Town, South Wollo Amhara State, Ethiopia, a similar finding emerged where women who had higher educational attainment were found to be more knowledgeable about MTCT of HIV Option B+ compared to their counterparts with low/no formal education.^14^

It further emerged from our study that women living with HIV in rural areas of Malawi were more likely to possess adequate knowledge compared to their counterparts in urban areas. This finding may seem to contrast with that of earlier studies elsewhere in Ethiopia, suggesting that women in urban areas have improved knowledge of MTCT of HIV due to better access to health resources, including healthcare facilities and health information.^25–27^ In respect of our findings in Malawi, we believe concerted efforts to increase women’s access to MTCT of HIV services in rural Malawi may have worked over time to increase women living with HIV in rural communities to have improved knowledge of MTCT of HIV relative to their urban counterparts.^28^ This finding calls for increased attention to designing and targeting women living with HIV in the urban settings of Malawi to enhance their knowledge of MTCT of HIV.

It also emerged from our study that women living with HIV who had adequate HIV knowledge and those that rejected the endorsement of HIV stigma and discrimination were more likely to possess adequate knowledge about MTCT of HIV compared to their counterparts who did not have adequate HIV knowledge and those that endorsed HIV stigma and discrimination. These findings underscore the importance of HIV health policy targeting all women, especially those living with HIV, with HIV prevention programs and strategies that seek to increase their clinical knowledge base of HIV transmission from person to person and MTCT of HIV. It is possible that women living with HIV who reject the endorsement of HIV stigma and discrimination have; first, access to credible sources of health information that help them to acquire an adequate clinical knowledge base of HIV transmission and MTCT of HIV which are useful for rejecting HIV discriminatory attitudes. Second, it is possible that women living with HIV who rejected HIV discriminatory attitudes are also more likely to seek medical treatment for their HIV infection. Through such treatment regimes, they get the opportunity to frequently interact with healthcare practitioners who provide them with health information on MTCT of HIV. In contrast, their counterparts holding HIV discriminatory attitudes may avoid healthcare spaces, limiting their opportunity to be informed about MTCT of HIV. Similar findings have been reported by Malaju and Alene^29^ who contend in Ethiopia that women with a lack of comprehensive knowledge about HIV transmission were less likely to able to fully digest the options available for preventing MTCT of HIV. Such women, the study further reported, also had limited access to spaces and services that help them prevent MTCT of HIV. Among pregnant women attending ANC in Ethiopia, Abtew and colleagues^30^ reported a similar finding where women’s comprehensive knowledge of MTCT of HIV was associated with having sufficient knowledge of HIV/AIDS.

Our study has some noteworthy limitations. First, our data is a bit dated and there may be a need to collect current data to understand these relationships. Second, the MDHS was collected contemporaneously, meaning our findings are limited to statistical association and must be interpreted cautiously. Finally, our study may not have been exhaustive in capturing all the factors that influence knowledge of MTCT of HIV among women living with HIV in Malawi. This calls for future studies to combine qualitative methods with other survey questionnaires to capture the exhaustive list of factors influencing women’s knowledge of MTCT of HIV. Despite these limitations, our study makes an important contribution to the literature and policy on MTCT of HIV as it is among the first to examine the determinants of knowledge of MTCT of HIV among women living with HIV in Malawi and SSA.

We make some policy suggestions based on our findings. We recommend the need for further studies, especially those using mixed qualitative and quantitative approaches, to unpack the most influential factors contributing to shaping the knowledge of MTCT of HIV among Malawian women living with HIV. This is particularly foundational to orienting HIV health policy in Malawi as the country strives to eliminate vertical transmission of HIV. It is particularly useful to design health information that is purposefully targeted at women living with HIV who have no formal education. This may include translating messages into local dialects and basic language for easy digestion and consumption. In addition, it may also be prudent to integrate MTCT of HIV prevention strategies with the economic empowerment of women as a way to remove financial barriers that prevent these women from interacting with healthcare spaces where they can learn about MTCT of HIV. In addition, women living with HIV in the Northern Region and urban areas of Malawi may require intensified policy attention to increase their exposure to clinical information on MTCT of HIV. Overall, there is an urgent need to offer women living with HIV in Malawi the opportunity to improve their knowledge of MTCT of HIV if the country is to make progress towards the elimination of MTCT of HIV as part of the overall strategy to contain new infections of HIV in the country.

## Data Availability

The Malawi 2015-16 DHS dataset is available at https://dhsprogram.com/data/dataset/Malawi_Standard-DHS_2015.cfm?flag=0
